# Assessing the Relationship between LAMS and CT Perfusion Parameters in Acute Ischemic Stroke Secondary to Large Vessel Occlusion

**DOI:** 10.3390/jcm12103374

**Published:** 2023-05-09

**Authors:** Karissa C Arthur, Shenwen Huang, Julie C. Gudenkauf, Alireza Mohseni, Richard Wang, Alperen Aslan, Mehreen Nabi, Meisam Hoseinyazdi, Brenda Johnson, Navangi Patel, Victor C Urrutia, Vivek Yedavalli

**Affiliations:** 1 Department of Neurology, Virginia Commonwealth University, Richmond, VA 23298, USA; 2 Department of Neurology, Johns Hopkins University, Baltimore, MD 21287, USA; 3 Russell H. Morgan Department of Radiology and Radiological Sciences, Johns Hopkins University School of Medicine, Baltimore, MD 21287, USA

**Keywords:** ischemic stroke, perfusion imaging, diagnosis, collateral status

## Abstract

Background: The Los Angeles Motor Scale (LAMS) is a rapid pre-hospital scale used to predict stroke severity which has also been shown to accurately predict large vessel occlusions (LVOs). However, to date there is no study exploring whether LAMS correlates with the computed tomography perfusion (CTP) parameters in LVOs. Methods: Patients with LVO between September 2019 and October 2021 were retrospectively reviewed and included if the CTP data and admission neurologic exams were available. The LAMS was documented based on emergency personnel exams or scored retrospectively using an admission neurologic exam. The CTP data was processed by RAPID (IschemaView, Menlo Park, CA, USA) with an ischemic core volume (relative cerebral blood flow [rCBF] < 30%), time-to-maximum (Tmax) volume (Tmax > 6 s delay), hypoperfusion index (HI), and cerebral blood volume (CBV) index. Spearman’s correlations were performed between the LAMS and CTP parameters. Results: A total of 85 patients were included, of which there were 9 intracranial internal carotid artery (ICA), 53 proximal M1 branch middle cerebral artery M1, and 23 proximal M2 branch occlusions. Overall, 26 patients had LAMS 0–3, and 59 had LAMS 4–5. In total, LAMS positively correlated with CBF < 30% (Correlation Coefficient (CC): 0.32, *p* < 0.01), Tmax > 6 s (CC:0.23, *p* < 0.04), HI (CC:0.27, *p* < 0.01), and negatively correlated with the CBV index (CC:−0.24, *p* < 0.05). The relationships between LAMS and CBF were < 30% and the HI was more pronounced in M1 occlusions (CC:0.42, *p* < 0.01; 0.34, *p* < 0.01 respectively) and proximal M2 occlusions (CC:0.53, *p* < 0.01; 0.48, *p* < 0.03 respectively). The LAMS also correlated with a Tmax > 6 s in M1 occlusions (CC:0.42, *p* < 0.01), and negatively correlated with the CBV index in M2 occlusions (CC:−0.69, *p* < 0.01). There were no significant correlations between the LAMS and intracranial ICA occlusions. Conclusions: The results of our preliminary study indicate that the LAMS is positively correlated with the estimated ischemic core, perfusion deficit, and HI, and negatively correlated with the CBV index in patients with anterior circulation LVO, with stronger relationships in the M1 and M2 occlusions. This is the first study showing that the LAMS may be correlated with the collateral status and estimated ischemic core in patients with LVO.

## 1. Introduction

Stroke is one of the leading causes of death and disability, with ischemic stroke being the most common [1]. Of those, ischemic strokes, due to large vessel occlusion (LVO), contribute disproportionally to post-stroke dependence and death [2]. Mechanical thrombectomy (MT) has proven to improve functional outcomes in patients with LVO [3], and earlier reperfusion has been associated with better outcomes [4]. Therefore, the ability to quickly recognize patients with LVO in the field would be beneficial and allow those patients to be taken directly to comprehensive stroke centers, decreasing the time to reperfusion, and improving outcomes [5,6]. Several rapid pre-hospital stroke scales, used by emergency personnel, have been shown to predict LVOs [7].

The Los Angeles Motor Scale (LAMS) is one such rapid pre-hospital scale that was developed to predict stroke severity [8] and is useful when considering whether patients should be diverted to comprehensive stroke centers. Studies have shown that the LAMS has a strong correlation with admission National Institutes of Health Stroke Scale (NIHSS) and is comparable, if not superior, to other utilized pre-hospital stroke scales [9]. Moreover, the LAMS has good accuracy in predicting LVOs [10]. An increased LAMS severity with a score of 4–5 double the likelihood of LVOs compared to a LAMS score of 0–3 [9].

A comprehensive baseline computed tomography (CT) imaging–comprised of non-contrast CT (NCCT), CT angiography (CTA), and CT perfusion (CTP)–is widely utilized for triaging patients presenting with acute ischemic stroke (AIS) secondary to anterior circulation LVO for MT. The addition of CTP to comprehensive baseline imaging increases the sensitivity for detecting ischemic core compared to NCCT only [11] while offering additional data in evaluating a patient’s collateral status (CS) and eligibility for MT [12]. Poor CS is associated with an increased risk of post-MT complication and overall worse functional outcomes [13,14]. The hypoperfusion index (HI) and cerebral blood volume (CBV) index are parameters obtained from CTP that correlate with the collateral status and infarct growth, and guide decisions for MT [15,16,17,18,19]. There are no current studies exploring the relationship between pre-hospital stroke scales and CTP parameters.

Our preliminary study aimed to explore the relationship between the LAMS score and baseline CTP parameters. Such a relationship may suggest a biological correlation between the LAMS and collateral status and ischemic core.

## 2. Methods

### 2.1. Population

The data for the study population was collected through the Johns Hopkins Hospital Comprehensive Stroke Center Database. This study was approved through the Johns Hopkins School of Medicine institutional review board (JHU-IRB00269637). In this retrospective multicenter analysis, we identified consecutive patients from 1 September 2019 to 1 April 2021 who underwent NCCT, CTA, and CTP with confirmed anterior circulation LVO on CTA (defined as distal intracranial internal carotid artery (ICA), M1, and proximal M2 segments of the middle cerebral artery (MCA). Patients were excluded from the analysis if CTP was obtained >24 h after admission or after a mechanical thrombectomy procedure or if patients were incorrectly classified as having LVO (e.g., internal carotid artery stenosis). 

### 2.2. Data Collection

Patients were included in the study if they had an LVO, CTP with available post-processed parameters, and a documented neurologic exam before the time of imaging. The baseline and clinical data collected for each patient included demographics, risk factors for AIS (including coronary artery disease, hypertension, hyperlipidemia, diabetes mellitus, atrial fibrillation, prior stroke, smoking status, and body mass index (BMI), a baseline LAMS score, and a site of occlusion. When the LAMS score or admission NIHSS data was unavailable, the scores were estimated based on admission neurologic exam and history [8]. The LAMS score ranged from 0 to 5 based on the addition of the following components: 0 or 1 for facial strength (normal or droop); 0, 1, or 2 for arm strength (normal, drifts down, falls rapidly), and 0, 1, or 2 for grip strength (normal, weak, no grip). Patients were stratified into the LAMS 0–3 versus 4–5 for the primary analysis [20]. The vessel-based subgroup analysis was also performed for distal intracranial ICA, M1, and proximal M2 segments respectively. 

### 2.3. Imaging

The NCCT was acquired on the Siemens Flash and/or Drive (Siemens Healthineers, Erlangen, Germany) with the following parameters: The helical mode at a 5 mm slice thickness (ST) (120 kVp, 365 mAs, rotation time 1 s, acquisition time 6–8 s, collimation 128 × 0.6 mm, pitch value 0.55, scan direction CC).

The CT ASPECTS score was obtained from documented experienced neuroimaging interpreters. If ASPECTS was unavailable, CT imaging was reviewed independently by a board-certified neuroradiologist to determine the ASPECTS score (VY, 6 years of experience).

The CTP was performed on a Siemens Flash and/or Drive (Siemens Healthineers, Erlangen, Germany) with the following parameters: The injection of 50 mL non-ionic iodinated contrast with 30 mL saline flush at 5–6 mL/s with a coverage of 70–100 mm at 5 mm slice thickness, 70 kVP, 200 effective mAs, rotation time 0.25 s, average acquisition time 60 s, collimation 48 × 1.2 mm, pitch value 0.7, 4D range 114 mm × 1.5 s. The CTP images are then post-processed using RAPID commercial software (IschemaView, Menlo Park, CA, USA) for generating Tmax maps. HI is then automatically calculated as a ratio of the calculated Tmax > 10 s delay volume divided by a Tmax > 6 s delay volume in mL (Tmax > 6 s) in the affected hemisphere. The ischemic core on the CTP is quantitatively estimated by the relative cerebral blood volume (rCBF) < 30% volume in mL (rCBF < 30%). Tissue at risk is defined as a Tmax > 6 s. Mismatch volume is defined as the difference between the Tmax > 6 s and the rCBF < 30%. The mismatch ratio is calculated as the Tmax > 6 s divided by the rCBF < 30%. The CBV index is automatically calculated by RAPID as the ratio of mean rCBV values within the Tmax >6 s region with the mean CBV in normal brain regions. The target mismatch profile for CTP is defined based on the prior study by Lansberg et al. [12]. 

### 2.4. Statistical Analysis

The collected data were coded, tabulated, and statistically analyzed using IBM SPSS statistics (Statistical Package for Social Sciences) software version 28.0, IBM Corp., Chicago, IL, USA, 2021. The quantitative data was described as mean ± SD (standard deviation) and then compared using an independent t-test after being tested for normality using the Shapiro-Wilk test. The qualitative data were described as numbers and percentages and compared using the Chi-squared test and Fisher’s Exact test for variables with small, expected numbers. The correlation coefficients (CC) of the LAMS with the CTP parameters were calculated using the Spearman correlation test. The level of significance was taken at *p*-value ≤ 0.05, otherwise was non-significant.

## 3. Results

Eighty-five patients were included. Twenty-eight (32.9%) were scored with a LAMS 0–3 with 57 (67.1%) in the LAMS 4–5 category. 

**Figure 1:**
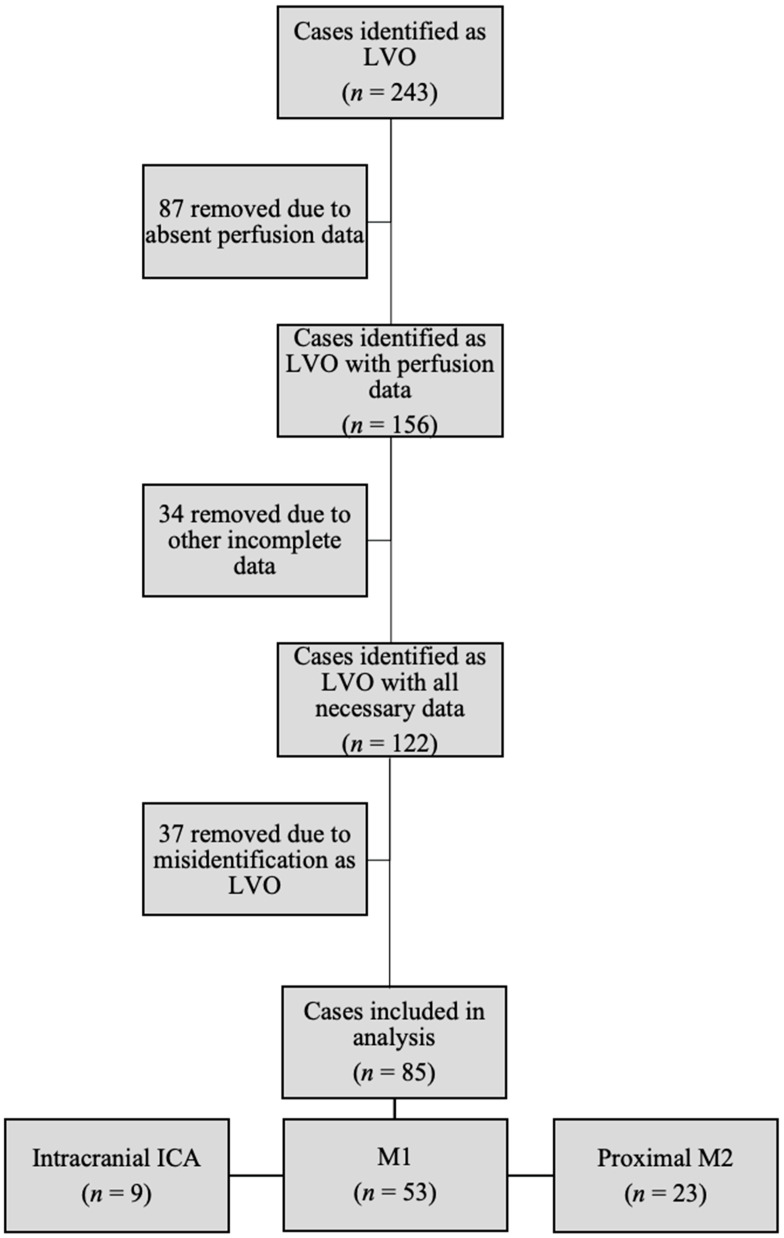
Flow chart of retrospective chart review to identify patients with LVO, admission neurologic exam, and perfusion data. LVO = large vessel occlusion; ICA = internal carotid artery; M1 = M1 branch of middle cerebral artery (MCA), M2 = M2 branch of MCA.

Eighty-five patients had LAMS, ASPECTS, and CTP parameters available for analysis (Figure 1). Patients with mismatch ratios of infinity were excluded from the LAMS mismatch ratio correlation, resulting in 46 out of 85 (54.1%) available for analysis of this parameter. In total, 84 out of 85 patients (98.8%) with a Tmax > 6 s and HI, and 66 out of 85 (77.6%) with CBV indices were available for review. Table 1 compares patients with a LAMS score of 0–3 to those with a score of 4–5. The groups were comparable demographically except for age, with patients scoring 4–5 being older (LAMS 0–3, 63.1 ± 14.23 years old versus LAMS 4–5, 71.02 ± 14.4 years old, *p* = 0.02). Please refer to Table 1 for additional details. 

The 85 patients included in the study were comprised of nine distal intracranial ICA (9/85, 10.5%); 53 M1 (62.3%); and 23 proximal M2 (23/85, 27.1%) occlusions. 

There were significant small to moderate positive correlations between the LAMS score and CBF <30% (mL) (CC 0.32, *p* < 0.01), Tmax > 6 s (CC:0.23, *p* < 0.04), and HI (CC:0.27, *p* < 0.01), and small negative correlation with the CBV index (CC:−0.24, *p* < 0.05) with the CBV index based on a post-processed analysis from a widely used commercial software platform (Table 2). There was no significant correlation between the LAMS score and imaging parameters when the intracranial ICA occlusions were isolated (Table 3). 

### 3.1. M1 Segmental Subgroup Analysis

In patients with M1 occlusions, the LAMS score was moderate to positively correlated with a CBF < 30% (CC:0.42, *p* < 0.01), Tmax > 6 s (CC:0.42, *p* < 0.01), and HI (0.34, *p* < 0.01), and negatively correlated with ASPECTS (CC:−0.30, *p* < 0.03) (Table 4).

### 3.2. Proximal M2 Segment Subgroup Analysis

In patients with proximal M2 occlusions, the LAMS score was moderate to strongly positively correlated with a CBF < 30% and HI (CC:0.53, *p* < 0.01; 0.48, *p* < 0.03 respectively). The LAMS score was strongly negatively correlated with the CBV index (CC:−0.69, *p* < 0.01) (Table 5). There was no correlation between the LAMS and ASPECTS scores in the M2 occlusions.

In summary, in patients with M1 or proximal M2 occlusions, the LAMS score is positively correlated with a CBF < 30% and HI. In patients with M2 occlusions, the LAMS score is further positively correlated with a Tmax > 6 s, and in patients with M1 occlusions, the LAMS is further negatively correlated with the ASPECTS score. In patients with intracranial ICA occlusions, no significant correlation between the LAMS score and the perfusion imaging parameters were demonstrated.

## 4. Discussion

We demonstrate that the LAMS score showed weak to moderate significant positive correlations with an rCBF < 30, a Tmax > 6 s, and HI in all patients with LVOs, though when affected vessels were analyzed separately, there were no correlations in patients with ICA occlusions. The results indicate that a rapid pre-hospital stroke scale may help determine which patients within the anterior circulation LVO subset also have a larger ischemic core and poor CS, an important biological correlation. After stratifying by the LVO segment (intracranial ICA, M1, and proximal M2), the results show that these relationships are most pronounced in MCA occlusions. There is no significant correlation between the LAMS score and the CTP imaging parameters in distal intracranial ICA occlusions, however, the sample size of patients with intracranial ICA occlusions was small. Future research should include a larger sample of patients with intracranial ICA occlusions to assess for a possible relationship. The positive correlation between the LAMS and HI index and the negative correlation of the CBV index specifically support the potential relationship between the LAMS and CS. 

Poor CS has been shown to be associated with a larger core infarct size on admission [21] and larger 24-h infarct volumes [22]. Further, poor CS is associated with a higher ischemic core growth rate, meaning that patients with poor CS have less time to save salvageable tissue [23]. Previous research has shown that patients with poor CS are more likely to have high admission NIHSS scores [13]. Our results are in line with the correlation between the admission NIHSS and the admission LAMS score [8]. The LAMS, however, can be completed more rapidly than the NIHSS by emergency personnel. Therefore, being able to use the LAMS score to potentially predict CS is important for the rapid triaging of patients to stroke centers where treatment can be offered. This may help to prioritize transport to a comprehensive stroke center rather than the nearest hospital due to the faster ischemic core growth rate in patients with poor CS [23].

The moderate to strong correlations between the LAMS and a CBF < 30% indicate that the LAMS may also predict an ischemic core. In addition, the admission NIHSS has been associated with an ischemic core volume, with a lower NIHSS correlating with a smaller ischemic core [24]. Our results are in line with the correlation between the admission NIHSS and the admission LAMS score [8]. Interestingly, there was no correlation between the LAMS and ASPECTS. Prior research has shown that a CT ASPECTS score does not predict functional outcomes, while the CTP ASPECTS using a threshold of CBF < 30% for the core is predictive of functional outcomes [25]. A simple stroke scale, which may predict a CBF < 30% and collateral status, may therefore provide more information than an ASPECTS score as to which patients will benefit from thrombectomy. This is further supported by research showing that patients, even with a low CT ASPECTS, benefit from thrombectomy [26,27].

There are several limitations to this preliminary study. First, the LAMS scores, when unavailable from EMS personnel, were obtained retrospectively based on admission exams. These exams were usually performed by the consulting neurologist as opposed to EMS personnel. However, the entry LAMS score has been shown to have good convergent validity with the admission NIHSS and admission physical exam when retroactively scored [8]. Patients with a LAMS score of 4–5 were older than patients with a LAMS score of 0–3, and studies have shown that age correlates with poor CS [28]. Therefore we may expect older patients to have a higher LAMS based on our results. A further limitation of this study was the small sample size and the restriction of the sample of stroke patients with LVO. This study is predicated on the accuracy of the LAMS predicting LVOs, but we cannot know with certainty at the time of the LAMS if the patient in fact has an LVO. Future studies should compare the accuracy of the LAMS in predicting LVOs and CS in all suspected stroke patients. Nevertheless, our study shows a good correlation with surrogates for CS in this population and, therefore, may suggest that the LAMS has a biological correlation with CS, especially in MCA occlusions. The data more robustly supports the practice of using the LAMS to detect LVO in the field, which is especially important when considering triage to a comprehensive stroke center [29]. This information would be further useful in patients presenting within 6 h of treatment onset, as it may be reasonable to bypass advanced imaging and go directly to the angiography suite in patients with a higher LAMS, given the risk of poor CS and early ischemia. This approach has been shown to decrease transfer times [30,31] and higher rates of clinical improvement at 24 h [31]. Finally, given the relationship between poor CS with worse outcomes in patients with LVOs [14,32,33,34,35], future research should explore the utility of the LAMS in predicting recanalization and overall clinical outcomes with a larger prospective study.

In summary, this is the first study exploring the relationship between a rapid pre-hospital stroke scale and cerebral perfusion parameters. The results show that the LAMS may be correlated with CS and the ischemic core in patients with LVO. More research needs to be performed to validate these results. Nevertheless, these results suggest that a simple pre-hospital scale such as the LAMS may have value beyond LVO detection by providing information on the ischemic core and CS at the time of presentation. Poor CS specifically is associated with higher rates of infarct growth [23], and patients with poor CS should be transported to a comprehensive stroke center quickly to improve functional outcomes by decreasing the time to reperfusion [5]. The ability of a rapid scale, such as the LAMS to predict CS, is therefore extremely useful in selecting patients in which primary stroke centers should be bypassed. Future studies should build on these results to assess the utility of the LAMS in triaging stroke patients in the field for the timely transfer to a comprehensive stroke center for appropriate intervention.

## Figures and Tables

**Table 1:** Descriptive statistics among the studied cases and comparison according to LAMS score.

Variables			All Cases (N = 85)	LAMS Score		*p*-Value
				0–3 (N = 28)	4–5 (N = 57)	
Age (years), Mean ± SD			68.6 ± 14.7	63.1 ± 14.23	71.02 ± 14.4	⌂ 0.02 *
Sex (*n*, %)	Male		40 (47.1%)	13 (50%)	27 (45.7%)	# 0.97
	Female		45 (52.9%)	13 (50%)	32 (54.23%)	
Race (*n*, %)	African		44 (51.8%)	14 (53.8%)	30 (50.84%)	§ 0.89
	White/Caucasian		36 (42.4%)	10 (38.4%)	26 (44.06%)	
	Others		5 (5.9%)	2 (7.6%)	3 (5.08%)	
Atrial Fibrillation, (*n*, %)			28 (32.9%)	6 (23.1%)	22 (37.3%)	# 0.86
Diabetes, (*n*, %)			25 (29.4%)	6 (23.1%)	19 (32.2%)	# 0.87
Dyslipidemia, (*n*, %)			30 (35.3%)	10 (38.5%)	20 (33.9%)	# 0.12
HTN, (*n*, %)			60 (70.6%)	14 (53.8%)	46 (78.0%)	# 0.14
Prior CVA, (*n*, %)			17 (20.0%)	4 (15.4%)	13 (22.0%)	§ 0.92
CKD, (*n*, %)			8 (9.4%)	4 (15.4%)	4 (6.8%)	§ 0.13
Smoking history, (*n*, %)			16 (18.8%)	4 (15.4%)	12 (20.3%)	§ 0.74
CAD, (*n*, %)			16 (18.8%)	6 (23.1%)	10 (16.9%)	§ 0.83
ASPECTS, Mean ± SD			7.4 ± 2.5	7.8 ± 2.4	7.3 ± 2.6	⌂ 0.41
Time parameters, minutes Median (IQR)		Last known well to door	230 (70, 642)	148 (74, 736)	262 (70, 592)	^ 0.98
		Door to CT	29 (18, 44)	28 (18, 37)	30 (21, 45)	^ 0.35
		Last known well to CT	274.5 (98, 683)	198 (87, 796)	302 (112, 635)	^ 0.88

§ Fishers Exact test. # Chi square test. ⌂ Independent t-test. ^ Mann-Whitney U test. * Significant (0.050). LAMS = Los Angeles Motor Scale; HTN = hypertension; CVA = cerebrovascular accident; CKD = chronic kidney disease, CAD = coronary artery disease; CT = computed tomography; IQR = interquartile range.

**Table 2:** Correlation between LAMS score with imaging parameters for all cases.

Total Cases (*n* = 85)	Descriptive (*n*)	*p* Value	Correlation Coefficient
CBF < 30% (mL)	24.94 ± 42.6(85)	0.01 *	0.32
Tmax > 6 s (mL)	113.61 ± 83.4(84)	0.04 *	0.23
Mismatch Volume (mL)	88.67 ± 77.7(84)	0.36	0.10
Mismatch ratio	6.34 ± 7.03(46)	0.98	−0.01
Hypoperfusion Index	0.375 ± 0.25(84)	0.01 *	0.27
ASPECTS	7.45 ± 2.57(85)	0.38	−0.10
CBV Index (rCBV Tmax > 6 s)	0.70 ± 0.19(66)	0.05 *	−0.24
LAMS score	3.85 ± 1.5(85)	0	1

* Significant (0.050). CBF = cerebral blood flow, CBV = cerebral blood volume.

**Table 3:** Correlation between LAMS with imaging parameters for intracranial ICA occlusions.

Intracranial ICA Only (*n* = 9)	Descriptive (*n*)	*p* Value	Correlation Coefficient
CBF < 30% (mL)	62.63 ± 82.66 (11)	0.81	0.08
Tmax > 6 s (mL)	200.81 ± 137.16 (11)	0.43	0.27
Mismatch Volume (mL)	138.18 ± 144.6 (11)	0.65	0.15
Mismatch ratio	4.43 ± 4.07 (8)	0.95	0.03
Hypoperfusion Index	0.55 ± 0.22 (11)	0.86	0.06
ASPECTS	5.18 ± 3.45 (11)	0.40	0.29
CBV Index (rCBV Tmax > 6 s)	0.655 ± 2.87 (9)	0.56	−0.23
LAMS score	3 ± 1.67 (11)	0	1

* Significant (0.050).

**Table 4:** Correlation between LAMS with imaging parameters for M1 occlusions.

M1 (*n* = 53)	Descriptive (*n*)	*p* Value	Correlation Coefficient
CBF < 30% (mL)	19.8 ± 31.4 (52)	0.01 *	0.42
Tmax > 6 s (mL)	113 ± 66.2 (52)	0.01 *	0.42
Mismatch Volume (mL)	93.8 ± 61.3 (52)	0.28	0.24
Mismatch ratio	7.98 ± 8.42 (27)	0.57	−0.12
Hypoperfusion Index	0.33 ± 0.25 (52)	0.01 *	0.34
ASPECTS	7.69 + 2.39 (52)	0.03 *	−0.30
CBV Index (rCBV Tmax > 6 s)	0.73 ± 0.14 (40)	0.39	−0.14
LAMS score	4.06 ± 1.29 (52)	0	1

* Significant (0.050).

**Table 5:** Correlation between LAMS score with imaging parameters for proximal M2 occlusion.

Proximal M2 (*n* = 23)	Descriptive (*n*)	*p* Value	Correlation Coefficient
CBF < 30% (mL)	17.7 ± 26.8 (21)	0.01 *	0.53
Tmax > 6 s (mL)	67.6 ± 43.9 (21)	0.69	0.09
Mismatch Volume (mL)	49.9 ± 45.1 (21)	0.21	−0.29
Mismatch ratio	3.72 ± 2.92 (11)	0.57	−0.20
Hypoperfusion Index	0.37 ± 0.25 (21)	0.03 *	0.48
ASPECTS	8.05 ± 1.88 (22)	0.53	−0.14
CBV Index (rCBV in Tmax > 6 s)	0.68 ± 0.23 (17)	0.01 *	−0.69
LAMS score	3.77 ± 1.77 (22)	0	1

* Significant (0.050).

## Data Availability

The data presented in this study are available on request from the corresponding author. The data are not publicly available due to privacy.

## References

[1] (2021). GBD 2019 Stroke Collaborators. Global, regional, and national burden of stroke and its risk factors, 1990–2019: A systematic analysis for the Global Burden of Disease Study 2019. Lancet Neurol..

[2] Malhotra K., Gornbein J., Saver J.L. (2017). Ischemic Strokes Due to Large-Vessel Occlusions Contribute Disproportionately to Stroke-Related Dependence and Death: A Review. Front. Neurol..

[3] Goyal M., Menon B.K., Van Zwam W.H., Dippel D.W., Mitchell P.J., Demchuk A.M., Dávalos A., Majoie C.B., van Der Lugt A., De Miquel M.A. (2016). Endovascular thrombectomy after large-vessel ischaemic stroke: A meta-analysis of individual patient data from five randomised trials. Lancet.

[4] Saver J.L., Goyal M., Van der Lugt A., Menon B.K., Majoie C.B., Dippel D.W., Campbell B.C., Nogueira R.G., Demchuk A.M., Tomasello A. (2016). Time to treatment with endovascular thrombectomy and outcomes from ischemic stroke: A meta-analysis. JAMA.

[5] Hong J.B., Diprose W.K., Wang M.T.M., Meyer J., Kilfoyle D., Brew S., Barber P.A. (2022). Bypass of Primary Stroke Centers Compared With Secondary Transfer for Endovascular Thrombectomy. Stroke Vasc. Interv. Neurol..

[6] Mohamad N.F., Hastrup S., Rasmussen M., Andersen M.S., Johnsen S.P., Andersen G., Simonsen C.Z. (2016). Bypassing primary stroke centre reduces delay and improves outcomes for patients with large vessel occlusion. Eur. Stroke J..

[7] Zhao H., Coote S., Pesavento L., Churilov L., Dewey H.M., Davis S.M., Campbell B.C.V. (2017). Large Vessel Occlusion Scales Increase Delivery to Endovascular Centers Without Excessive Harm From Misclassifications. Stroke.

[8] Llanes J.N., Kidwell C.S., Starkman S., Leary M.C., Eckstein M., Saver J.L. (2004). The Los Angeles Motor Scale (LAMS): A new measure to characterize stroke severity in the field. Prehospital Emerg. Care.

[9] Noorian A.R., Sanossian N., Shkirkova K., Liebeskind D.S., Eckstein M., Stratton S.J., Pratt F.D., Conwit R., Chatfield F., Sharma L.K. (2018). Los Angeles Motor Scale to Identify Large Vessel Occlusion. Stroke.

[10] Nazliel B., Starkman S., Liebeskind D.S., Ovbiagele B., Kim D., Sanossian N., Ali L., Buck B., Villablanca P., Vinuela F. (2008). A brief prehospital stroke severity scale identifies ischemic stroke patients harboring persisting large arterial occlusions. Stroke.

[11] Shen J., Li X., Li Y., Wu B. (2017). Comparative accuracy of CT perfusion in diagnosing acute ischemic stroke: A systematic review of 27 trials. PLoS ONE.

[12] Lansberg M.G., Christensen S., Kemp S., Mlynash M., Mishra N., Federau C., Tsai J.P., Kim S., Nogueria R.G., Jovin T. (2017). Computed tomographic perfusion to Predict Response to Recanalization in ischemic stroke. Ann. Neurol..

[13] Fanou E.M., Knight J., Aviv R.I., Hojjat S.P., Symons S.P., Zhang L., Wintermark M. (2015). Effect of Collaterals on Clinical Presentation, Baseline Imaging, Complications, and Outcome in Acute Stroke. Am. J. Neuroradiol..

[14] Souza L.C.S., Yoo A.J., Chaudhry Z.A., Payabvash S., Kemmling A., Schaefer P.W., Hirsch J.A., Furie K.L., González R.G., Nogueira R.G. (2012). Malignant CTA collateral profile is highly specific for large admission DWI infarct core and poor outcome in acute stroke. AJNR Am. J. Neuroradiol..

[15] Guenego A., Marcellus D.G., Martin B.W., Christensen S., Albers G.W., Lansberg M.G., Marks M.P., Wintermark M., Heit J.J. (2019). Hypoperfusion Intensity Ratio Is Correlated With Patient Eligibility for Thrombectomy. Stroke.

[16] Guenego A., Fahed R., Albers G.W., Kuraitis G., Sussman E.S., Martin B.W., Marcellus D.G., Olivot J.M., Marks M.P., Lansberg M.G. (2020). Hypoperfusion intensity ratio correlates with angiographic collaterals in acute ischaemic stroke with M1 occlusion. Eur. J. Neurol..

[17] MacLellan A., Mlynash M., Kemp S., Ortega-Gutierrez S., Heit J.J., Marks M.P., Lansberg M.G., Albers G.W. (2022). Perfusion Imaging Collateral Scores Predict Infarct Growth in Non-Reperfused DEFUSE 3 Patients. J. Stroke Cerebrovasc. Dis..

[18] Arenillas J.F., Cortijo E., García-Bermejo P., Levy E.I., Jahan R., Liebeskind D., Goyal M., Saver J.L., Albers G.W. (2018). Relative cerebral blood volume is associated with collateral status and infarct growth in stroke patients in SWIFT PRIME. J. Cereb. Blood Flow Metab..

[19] Mlynash M., Lansberg M.G., Kemp S., Christensen S., Yennu A., Heit J.J., Marks M.P., Albers G.W. (2019). Abstract WP79: Combination of Tmax and Relative CBV Perfusion Parameters More Accurately Predicts CTA Collaterals Than a Single Perfusion Parameter in DEFUSE 3. Stroke.

[20] Stead T.G., Banerjee P., Ganti L. (2023). Real-World Field Performance of the Los Angeles Motor Scale as a Large Vessel Occlusion Screen: A Prospective Muticentre Study. Cerebrovasc. Dis..

[21] Xia Q., Wang X., Zhang Z., Fang Q., Hu C. (2019). Relationship between CT angiography-derived collateral status and CT perfusion-derived tissue viability. Clin. Radiol..

[22] Rao V.A.-O., Mlynash M., Christensen S., Yennu A., Kemp S., Zaharchuk G., Heit J.A.-O., Marks M.P., Lansberg M.G., Albers G.W. (2020). Collateral status contributes to differences between observed and predicted 24-h infarct volumes in DEFUSE 3. J. Cereb. Blood Flow Metab..

[23] Lin L., Yang J., Chen C., Tian H., Bivard A., Spratt N.J., Levi C.R., Parsons M.W. (2021). Association of Collateral Status and Ischemic Core Growth in Patients with Acute Ischemic Stroke. Neurology.

[24] Cao W., Ling Y., Yang L., Wu F., Zhang H., Cheng X., Dong Q. (2021). Association of Admission NIHSS Score with Infarct Core Volume and Target Mismatch of Infarct Core/Penumbra Volume on CT Perfusion in Acute Ischaemic Stroke. Cerebrovasc. Dis..

[25] Demeestere J., Scheldeman L., Cornelissen S.A., Heye S., Wouters A., Dupont P., Christensen S., Mlynash M., Albers G.W., Lansberg M. (2018). Alberta Stroke Program Early CT Score Versus Computed Tomographic Perfusion to Predict Functional Outcome after Successful Reperfusion in Acute Ischemic Stroke. Stroke.

[26] Huo X., Ma G., Tong X., Zhang X., Pan Y., Nguyen T.N., Yuan G., Han H., Chen W., Wei M. (2023). Trial of Endovascular Therapy for Acute Ischemic Stroke with Large Infarct. N. Engl. J. Med..

[27] Sarraj A., Hassan A.E., Abraham M.G., Ortega-Gutierrez S., Kasner S.E., Hussain M.S., Chen M., Blackburn S., Sitton C.W., Churilov L. (2023). Trial of Endovascular Thrombectomy for Large Ischemic Strokes. N. Engl. J. Med..

[28] Wiegers E.J.A., Mulder M.J.H.L., Jansen I.G.H., Venema E., Compagne K.C.J., Berkhemer O.A., Emmer B.J., Marquering H.A., van Es A.C.G.M., Sprengers M.E. (2020). Clinical and Imaging Determinants of Collateral Status in Patients with Acute Ischemic Stroke in MR CLEAN Trial and Registry. Stroke.

[29] Haight T., Tabaac B., Patrice K.-A., Phipps M.S., Butler J., Johnson B., Aycock A., Toral L., Yarbrough K.L., Schrier C. (2021). The Maryland Acute Stroke Emergency Medical Services Routing Pilot: Expediting Access to Thrombectomy for Stroke. Front. Neurol..

[30] Jadhav A.P., Kenmuir C.L., Aghaebrahim A., Limaye K., Wechsler L.R., Hammer M.D., Starr M.T., Molyneaux B.J., Rocha M., Guyette F.X. (2017). Interfacility Transfer Directly to the Neuroangiography Suite in Acute Ischemic Stroke Patients Undergoing Thrombectomy. Stroke.

[31] Ribo M., Boned S., Rubiera M., Tomasello A., Coscojuela P., Hernández D., Pagola J., Juega J., Rodriguez N., Muchada M. (2018). Direct transfer to angiosuite to reduce door-to-puncture time in thrombectomy for acute stroke. J. Neurointerv. Surg..

[32] Broocks G., Kemmling A., Meyer L., Nawabi J., Schön G., Fiehler J., Kniep H., Hanning U. (2019). Computed Tomography Angiography Collateral Profile Is Directly Linked to Early Edema Progression Rate in Acute Ischemic Stroke. Stroke.

[33] Lee T.J., Roh H.G., Kim J.H., Lee S.B., Park J.J., Lee H.J., Jeon Y.S., Choi J.W., Chun Y.I., Jung Y.J. (2021). Collateral and permeability imaging derived from dynamic contrast material-enhanced MR angiography in prediction of PH 2 hemorrhagic transformation after acute ischemic stroke: A pilot study. Neuroradiology.

[34] Feng X., Ye G., Cao R., Qi P., Lu J., Chen J., Wang D. (2020). Identification of Predictors for Hemorrhagic Transformation in Patients with Acute Ischemic Stroke After Endovascular Therapy Using the Decision Tree Model. Clin. Interv. Aging.

[35] Huang X., Yang Q., Shi X., Xu X., Ge L., Ding X., Zhou Z. (2019). Predictors of malignant brain edema after mechanical thrombectomy for acute ischemic stroke. J. Neurointerv. Surg..

